# PBPK Modeling of Acetaminophen in Pediatric Populations: Incorporation of SULT Enzyme Ontogeny to Predict Age-Dependent Metabolism and Systemic Exposure

**DOI:** 10.3390/life15071099

**Published:** 2025-07-13

**Authors:** Sonia Sharma, David R. Taft

**Affiliations:** Samuel J. and Joan B. Williamson Institute for Pharmacometrics, Division of Pharmaceutical Sciences, Arnold & Marie Schwartz College of Pharmacy and Health Sciences, Long Island University, Brooklyn, NY 11201, USA; sonia.sharma5@my.liu.edu

**Keywords:** PBPK modeling, acetaminophen, pediatrics, SULT, ontogeny, neonates

## Abstract

Sulfotransferase (SULT) enzymes contribute significantly to drug metabolism in pediatric patients. The purpose of this study was to develop a PBPK model for acetaminophen (APAP) in pediatric populations that accounts for the ontogeny of SULT isozymes that play a critical role in APAP metabolism. PBPK modeling and simulation were performed using the Simcyp^®^ Simulator. The model incorporated the developmental ontogeny of three key hepatic SULT enzymes: SULT1A1, SULT1A3, and SULT2A1 using “best-fit” ontogeny equations for each isozyme as determined by nonlinear regression analysis of enzyme abundance versus age. PBPK model-simulated pharmacokinetic profiles for APAP captured observed clinical data for systemic exposure (Cmax, AUC) in neonates, infants, and children. SULTS accounted for ~60% APAP metabolism in neonates, with decreased contributions to infants and children. Model sensitivity analysis highlighted the potential for APAP metabolic DDIs, primarily through SULT1A1. The study demonstrates that the impact of SULT enzymes on drug metabolism is significant in neonates, which is an important clinical consideration for APAP. A PBPK model that incorporates SULT ontogeny has the potential to help inform dosing decisions in this special patient population.

## 1. Introduction

Pediatric patients in intensive care units are particularly susceptible to potential drug-drug interactions (pDDIs) due to high rates of polypharmacy, influenced by complex pharmacotherapeutic regimens and physiological variations that affect drug metabolism and disposition. Studies have shown that the prevalence of pDDIs in these settings can be substantial, with significant risks for adverse drug events and therapy failures [1,2]. This highlights the critical need for careful medication management in pediatric patients, including consideration of any pharmacokinetic differences between the pediatric and adult populations. Pediatric subjects, particularly neonates and infants, are known to exhibit significant variability in drug metabolism and disposition from adults due to the immature state of drug-metabolizing enzymes and transporters (DMETs). Understanding the ontogeny of these DMETs is crucial for accurate drug dosing and minimizing adverse drug events in this vulnerable population [3]. Acetaminophen (N-(4-hydroxyphenyl) acetamide) (APAP) is widely used in infants and children to treat pain and fever. It is metabolized predominantly in the liver by multiple enzymatic pathways, including glucuronidation, mediated by UDP-glucuronosyltransferase (UGTs), and sulfate conjugation, mediated by cytosolic sulfotransferases (SULTs). Cytosolic SULTs are crucial for the biotransformation of endogenous molecules and xenobiotics, including albuterol and furosemide [1,4,5]. Although our understanding of SULTs is still evolving, this enzyme family is likely to play a crucial role in drug metabolism and has the potential for significant drug-drug interactions [6]. Furthermore, expression of SULT isozymes changes during the developmental stages from preterm to adulthood [7,8]. Published studies suggest that SULT activity is higher than UGT activity in children, highlighting the importance of SULT metabolism to pediatric pharmacokinetics. Correspondingly, the APAP glucuronide to sulfate metabolite ratio increased from 0.34 in newborns to 0.75 in children [9,10].

Physiologically based pharmacokinetic (PBPK) modeling has evolved as an indispensable tool in drug development, particularly for predicting drug-drug interactions (DDIs) and individualizing dosing strategies in special populations. Recent advancements have extended PBPK applications beyond cytochrome P450 (CYP) enzymes, addressing drugs cleared by non-CYP enzymes such as sulfotransferases (SULTs), which are pivotal in pediatric pharmacokinetics. Given the unique metabolic profiles and developmental changes of pediatric patients, this extension is crucial [11].

Ladumor et al. [7] conducted a comprehensive study on the ontogeny of SULT enzymes (SULT1A1, SULT1A3, SULT1B1, and SULT2A1), utilizing LC-MS/MS proteomic analysis across diverse age groups to quantify the age-dependent expression profiles of these enzymes in human liver. Their findings revealed significant variation in SULT enzyme abundance with age, ethnicity, and genotype, providing a foundational age-dependent ontogeny model for SULTs, which was applied within a PBPK model framework to predict age-related shifts in acetaminophen metabolism. The present investigation aims to extend this work by developing a PBPK model for APAP across pediatric populations, from neonates to children, that accounts for the ontogeny of SULT isozymes that play a critical role in APAP metabolism.

The ontogeny of SULT enzymes has been increasingly characterized over the past two decades, with studies showing that SULT1A3 exhibits high expression in fetal tissues that declines postnatally, while SULT1A1 and SULT2A1 display low neonatal expression followed by postnatal increases that peak during early childhood [12,13,14]. These developmental patterns are highly relevant to pediatric drug metabolism, particularly in the neonatal period when SULT activity may predominate over other conjugative pathways. While SULT-mediated drug-drug interactions (DDIs) are less commonly reported than those involving CYP enzymes, several compounds such as meclofenamic acid and dopamine are known inhibitors of specific SULT isozymes, including SULT1A1 and SULT1A3, raising the possibility of age-dependent DDI susceptibility [6,15].

Correspondingly, various modeling approaches have been used to characterize APAP pharmacokinetics across pediatric populations. Jiang et al. modeled pediatric acetaminophen disposition using lumped metabolic pathways and focused primarily on optimizing systemic PK predictions across age groups [16]. Cook et al. developed a Population Pharmacokinetic model for APAP in preterm and term neonates [17], but the model did not incorporate detailed ontogeny for individual SULT enzymes. Olafuyi et al. created a comprehensive PBPK model spanning preterm neonates to adolescents and incorporated ontogeny scaling for key metabolic pathways, including sulfation, based on published literature [18]. However, the implementation of SULT ontogeny relied on scaling assumptions and did not involve isozyme-specific regression modeling from raw enzyme abundance data. The current study builds upon these published models by including nonlinear regression-based ontogeny equations for SULT1A1, SULT1A3, SULT1B1, and SULT2A1, based on proteomic expression data.

The primary objective of this study is to develop and validate a pediatric PBPK model incorporating newly derived SULT ontogeny equations, thereby enabling the accurate prediction of APAP metabolism across neonatal, infant, and child populations. Additionally, model sensitivity analyses were conducted to evaluate the potential clinical implications of SULT-mediated DDIs on APAP systemic exposure in pediatric patients, highlighting the necessity for age-specific dosing strategies to optimize therapeutic outcomes and minimize adverse events in pediatric care.

## 2. Materials and Methods

### 2.1. Mathematical Characterization of SULT Ontogeny

An unweighted regression analysis was used to model the ontogeny of sulfotransferase isozymes (SULT1A1, SULT1A3, SULT1B1, and SULT2A1) using raw protein abundance data measured across postmenstrual age from neonates to adults, based on published quantitative proteomic studies [7]. The raw data was provided by Professor Bhagwat Prasad, University of Cincinnati (personal communication). All individual data points were included directly, without applying age-based weighting or stratification, to preserve full interindividual variability across the developmental spectrum. Several functional forms were evaluated for each SULT isozyme—including exponential, Hill-type (sigmoid), and logistic models—usingPython 3.8.17 and the “scipy.optimize.curve_fit” to minimize the unweighted residual sum of squares. Model performance was assessed through visual inspection of observed versus predicted data and residual error plots. The Akaike Information Criterion (AIC) guided the final model selection.

The final models for each SULT isozyme were piecewise functions, in which phase boundaries (breakpoints) were determined empirically by the best statistical fit to the data. These boundaries reflect inflection points in enzyme abundance trajectories and were not set a priori to match conventional pediatric age groups. This approach allows the ontogeny models to capture distinct biological transitions in enzyme expression, as supported by the raw data.

### 2.2. Physiologically-Based Pharmacokinetic (PBPK) Modeling

PBPK modeling was performed using Simcyp^®^ Simulator v23 (Certara, Sheffield, UK). The Healthy Volunteer and Pediatric Populations in Simcyp were modified to include the ontogeny equations for SULT1A1, SULT1A3, and SULT2A1. For SULT1E1, a constant ontogeny profile was assumed.

The Simcyp^®^ pediatric population models incorporate age-dependent physiologic and anatomic parameters, including organ weights, liver volume, hepatic and renal blood flow, glomerular filtration rate (GFR), plasma protein concentrations, and body composition, which collectively influence drug absorption, distribution, metabolism, and elimination in children. These parameters are dynamically scaled according to the virtual subjects’ age and are based on validated population datasets within the Simcyp framework. No further manual modifications were made to these default physiological settings, except for incorporation of the customized SULT ontogeny equations. A compound file for APAP was obtained from a published study by Olafuiy et al. [18], although two parameters (Vss and Kp scalar) were optimized in this research Model parameters are provided in Table 1. A full PBPK model was utilized, and Michaelis-Menten parameters were included for the relevant CYP (1A2, 2C9, 2C19, 2D6, 2E1, 3A4), UGT (1A1, 1A9, 2B15), and SULT (1A1, 1A3, 2A1,1E1) isozymes that mediate APAP metabolism.

Simulations were first conducted in the Healthy Volunteer population (n = 100 virtual subjects), with APAP administered as an IV infusion (2 h infusion) at 5 mg/kg and 20 mg/kg doses. Model verification employed visual predictive checks (VPCs), comparing simulated plasma concentration-time profiles to clinical data from a published study [19]. Those data were digitized using WebPlotDigitizer [20]. Predictive accuracy was assessed via fold-error analysis for maximum plasma concentration (Cmax) and area under the plasma concentration-time curve (AUC_0–∞_), with acceptable performance defined as fold errors between 0.5 and 2.0.

The verified PBPK model was then extended to the Pediatric population, with simulations performed in neonates (birth–28 days), infants (29 days–2 years), and children (2–12 years). Adolescents and adults were excluded from the primary analysis, as both groups exhibit similar SULT ontogeny profiles and acetaminophen pharmacokinetics, indicating that metabolic pathways are fully mature. The focus of this study is on pediatric age groups with the most pronounced developmental differences in SULT expression and drug disposition.

APAP was administered on a multiple IV dosing schedule (12.5 mg/kg infused over 15 min) every 6 h (neonates) or every 4 h (infants and children). Simulations were carried out for 48 hr after initiation of therapy, and model results were compared to published clinical data in each cohort. The observed plasma concentration-time profiles were digitized from a published study by Zuppa et al. [21] using WebPlot Digitizer [20].

Ten trial simulations were performed based on the number of subjects tested in the clinical study for each group: neonates (10 trials × two subjects, n = 20), infants (10 trials × 13 subjects, n = 130), and children (10 trials × nine subjects, n = 90). Model verification was performed as previously described.

### 2.3. Sensitivity Analysis

Sensitivity analyses were conducted to evaluate how changes in Michaelis–Menten parameters (Km and Vmax) for major SULT enzymes influenced APAP systemic exposure (C_max_ and AUC_0–∞_), a surrogate for pDDIs. For each isozyme, Km was increased over a range 10-fold greater than baseline (10 steps, log-distributed) to assess the potential impact of competitive inhibition. For Vmax, sensitivity analyses were performed over a ±10-fold range from baseline (20 steps, log-distributed), reflecting enzyme induction and mechanism-based inhibition scenarios. This analysis was conducted across pediatric subpopulations.

## 3. Results

### 3.1. SULT Ontogeny Modeling

**Figure 1:**
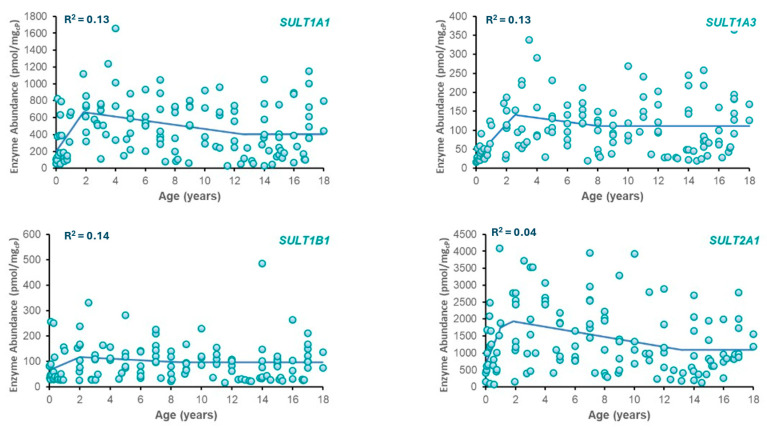
Plots of enzyme abundance vs. postnatal age for SULT isozymes. Included in each plot are observed data (circles) and model-predicted SULT enzyme abundance (solid line) using the equations in Table 2.

Best-fit ontogeny equations were determined for SULT enzymes (SULT1A1, SULT1A3, SULT2A1, and SULT1B1) based on available abundance data across ages (Table 2). Plots of SULT abundance vs. age are provided in Figure 1. From the models tested, the best fit was obtained with a three-phase piecewise linear function for each enzyme due to its superior ability to represent distinct developmental transitions, including neonatal peaks and stabilization during adulthood. The resulting phase boundaries did not necessarily align with conventional clinical age bins (e.g., <2 years, 2–12 years) but were instead defined by points of significant change in observed enzyme abundance. This data-driven method provides a more accurate reflection of the developmental kinetics of each SULT isozyme. While the piecewise linear model provided the best fit based on AIC, the considerable inter-individual variability typical of pediatric enzyme abundance datasets impacted the overall fit to the data as reflected in R^2^ values.

### 3.2. PBPK Model Verification

#### 3.2.1. Simulations in Healthy Volunteers

**Figure 2:**
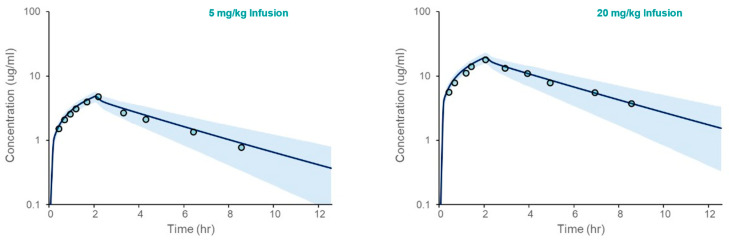
Acetaminophen PBPK model verification in an adult Healthy Volunteer population following IV administration. The plots depicted simulated (solid line) and observed (open circles) mean plasma concentrations vs. time following IV administration at two doses (5 and 20 mg/kg infused over 2 h). The shaded area spans the 5th and 95th percentiles for concentrations.

The PBPK model for acetaminophen was first verified in Healthy Volunteers. The results, presented in Figure 2, demonstrate that the model accurately captures clinical observations of the simulated mean plasma concentration over time following intravenous administration. The fold errors for model-predicted Cmax and AUC ranged from 1.04 to 1.24 (Table 3).

#### 3.2.2. Model Simulations in Pediatric Population

**Figure 3:**
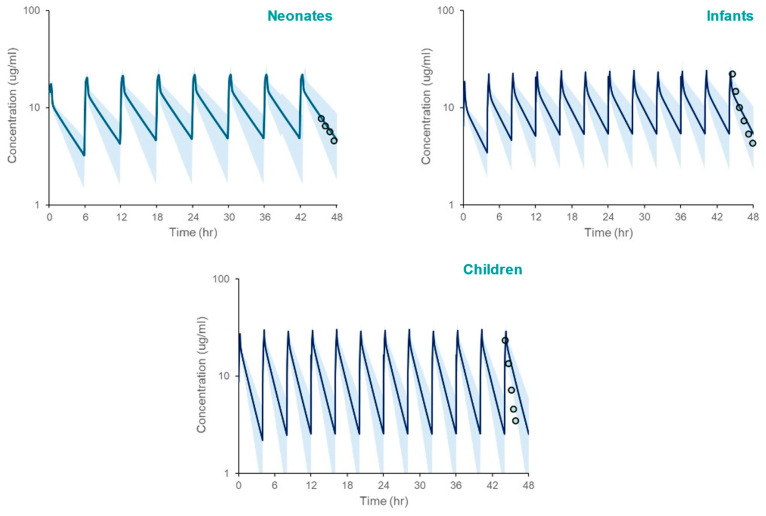
PBPK model simulated systemic exposure profiles for acetaminophen in neonates, infants, and children following repeated administration over 48 h. The shaded area spans the 5th and 95th percentiles for concentrations. The circles represent clinically measured plasma concentrations [21] using WebPlotDigitizer [20], reflecting the 42–48 h post-infusion interval after the last administered dose.

The PBPK model, incorporating SULT ontogeny equations, accurately predicted acetaminophen pharmacokinetics across pediatric populations. Simulated systemic exposure parameters (Cmax, AUC) closely matched observed clinical data. (Table 4, Figure 3), with fold errors ranging from 0.82 to 1.18. Although model simulations appear to overpredict the observed mean APAP systemic exposure in children based on Figure 2, that is not supported by the verification data in Table 4.

### 3.3. Model Sensitivity Analysis

**Figure 4:**
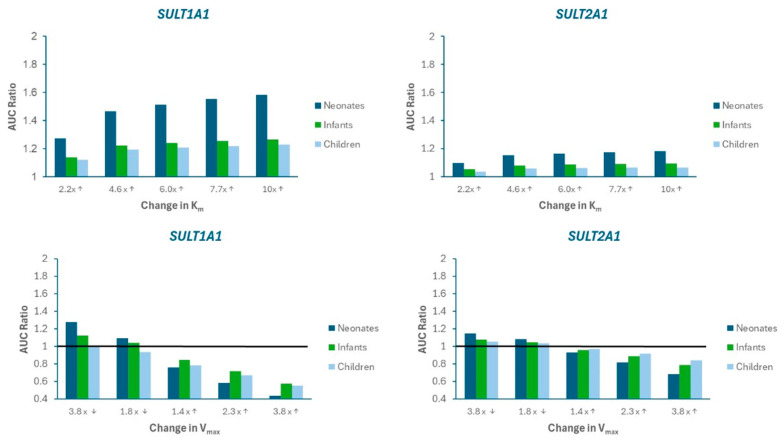
Sensitivity analysis illustrating the impact of changes in Michaelis-Menten parameters (K_m_, V_max_) on APAP AUC Ratio in neonates, infants, and children. AUC Ratio is calculated as the predicted AUC relative to baseline model values. The arrows on the labels on the x-axis indicate whether the parameter value was increased (↑) or decreased (↓). The horizontal black line represents the “no changet” boundary (AUC Ratio = 1).

A sensitivity analysis was performed to evaluate the impact of alterations in SULT metabolism kinetics on APAP systemic exposure. Figure 4 presents the changes in AUC ratio for SULT1A1 and SULT2A1 as Km is increased (imitating competitive inhibition) or V_max_ is decreased (imitating mechanism-based inhibition) and increased (imitating induction). Supplemental Figure S1 presents the mpact on Cmax ratio. Limited sensitivity was found for SULT1A3).

**Figure 5:**
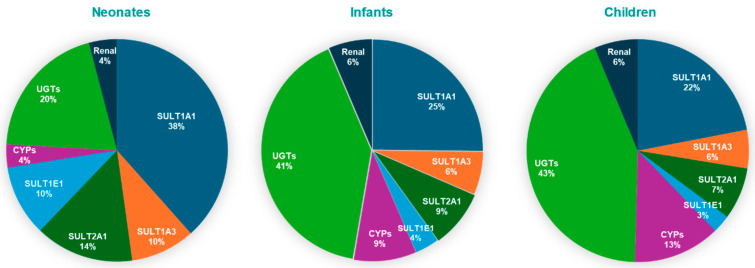
Pie charts depicting contributions of SULT isozymes, CYP, UGT, and renal excretion to APAP clearance in pediatric populations.

The results indicate that SULT1A1 is more susceptible to metabolic pDDIs, and that the impact is greater in neonates compared to other pediatric populations. These findings are consistent with a greater contribution of SULT1A1 to APAP metabolism, especially for neonates (Figure 5)

## 4. Discussion

PBPK modeling is recognized as a valuable tool in pediatric pharmacotherapy, as it informs dose selection, predicts drug-drug interactions (DDIs), and identifies knowledge gaps [22]. The current investigation builds upon published research on PBPK modeling of APAP in pediatric patients by incorporating the ontogeny of SULT isozymes that play an important role in APAP metabolism in this special population.

The developed ontogeny models for SULT enzymes effectively capture key biological trends in age-dependent enzyme expression. This is consistent with known developmental variability in enzyme activity, particularly in pediatric pharmacokinetics. Such variability, although challenging for model fitting, is well-recognized in the literature and supports the interpretability of moderate-fit models when applied to PBPK applications [23,24,25].

The regression analysis comparing candidate ontogeny models (exponential, sigmoidal/Hill, logistic, piecewise linear, and three-phase piecewise) revealed overall low R^2^ values (≤0.13), with the three-phase piecewise model demonstrating the best, albeit modest, fit. This finding reflects the high degree of inter-individual variability present in the raw enzyme abundance data, a phenomenon well-documented in pediatric enzyme ontogeny studies [26,27]. Despite this variability, the three-phase piecewise model was selected for its ability to capture key developmental transitions and provide biologically interpretable inflection points. However, the statistical limitations of this fit are acknowledged, and the implications for model predictions are discussed below.

The ontogeny profiles captured in the models align with previous studies. For example, SULT1A3 demonstrates high prenatal expression that declines postnatally, while SULT2A1 and SULT1B1 show postnatal increases [12,13,28]. These expression patterns reflect previously reported findings [8,22], confirming the biological relevance of the models. Moreover, these models serve as a foundation for predicting the age-specific metabolism of drugs, such as APAP, and highlight the importance of enzyme ontogeny in pediatric PBPK modeling.

Age-dependent enzyme contributions derived from our PBPK simulations validate the model’s physiological relevance. In the fetal liver, SULT1A3/1A4 is the primary sulfotransferase responsible for acetaminophen (APAP) conjugation [15,29]. Postnatally, SULT1A3 expression declines, while SULT1A1(a phenol sulfotransferase) becomes the dominant sulfating enzyme, accounting for approximately 38% of APAP clearance in neonates according to our model. This trend is supported by proteomic studies, which indicate that hepatic SULT1A1 levels are low at birth (~24% of adult levels) but rise rapidly during infancy, whereas SULT1A3 remains relatively constant or declines [7,30]. Thus, SULT1A1 is the primary enzyme mediating APAP sulfation in neonates, with smaller contributions from SULT1A3, SULT2A1, and SULT1E1.

As the infant matures, UGT-mediated glucuronidation increases markedly, gradually overtaking sulfation. Clinical pharmacokinetic data show that the glucuronide-to-sulfate metabolite ratio increases from ~0.34 in neonates to ~0.75 by early childhood [10], indicating a rise in glucuronide conjugates from 15–30% to nearly 43% of APAP metabolites. Our PBPK model reflects this trend, with UGT contributions rising from 20% in neonates to over 43% in children. Similarly, it was demonstrated that glucuronide formation clearance increases with age, while sulfate formation plateaus during infancy [31]. By early childhood, glucuronidation surpasses sulfation as the primary route of elimination. In parallel, CYP-mediated oxidation (e.g., via CYP2E1) also increases with age, although it remains a minor metabolic pathway. CYP2E1 is virtually absent at birth, rendering oxidative metabolism negligible in neonates [8,17,30].

These ontogenic patterns are further supported by in vitro and proteomic studies, which show that while sulfotransferases (particularly SULT1A1 and SULT2A1) are relatively well developed at birth, UGT enzymes exhibit delayed maturation [7,32,33,34]. Pediatric PBPK models integrating enzyme ontogeny profiles confirm that sulfation is the dominant clearance mechanism in neonates (~65–68% of APAP metabolism), but this fraction declines with age, whereas glucuronidation increases from ~30% in neonates to ~55–65% in adults [16,18]. Collectively, these findings support our model’s prediction that UGT-mediated glucuronidation becomes the predominant metabolic pathway for APAP by early childhood.

Model performance was evaluated through simulation of acetaminophen pharmacokinetics, which showed good agreement with clinical data. Simulated values for systemic exposure (Cmax, AUC) fell within the 95% confidence interval of observed data, and fold error values remained within acceptable predictive limits (0.5–2.0). These results underscore the practical utility of incorporating age-specific ontogeny into PBPK models to inform pediatric dosing strategies.

The pDDI sensitivity analysis simulations highlight the heightened sensitivity of pediatric patients, particularly neonates, to perturbations in SULT activity, with a significant impact via SULT1A1. Modulations in K_m_ and V_max_ led to substantial changes in AUC in these age groups, indicating that, because of their immature enzyme systems, newborns are particularly vulnerable to changes in enzyme affinity or catalytic capacity. These findings are in agreement with those by Cheung et al. [35] and Horace and Ahmed [36], who emphasized the need for cautious dosing in neonates receiving multiple medications.

Despite the growing recognition of the importance of SULTS to drug metabolism, there is limited information about known perpetrators of SULTS, including SULT1A1. While meclofenamate is recognized as a potent inhibitor of SULT1A1 and could theoretically serve as a mechanistic probe for PBPK model DDI simulations with APAP as a victim, it is not indicated for children under 14 years old [37]. As a result, there are no clinical studies of evaluating SULT-mediated DDI with APAP in children, and even in adults as such interactions are rarely reported or quantified. For this reason, model sensitivity analysis to simulate potential SULT-mediated DDIs in this study. However, it is likely that the analysis would be confirmed by PBPK model simulations using meclofenamate, a SULT1A1 perpetrator. However, these simulations would have limited clinical relevance to neonates and infants, the pediatric sub-populations where SULT metabolism is dominant.

Likewise, it was beyond the scope of this research to validate the PBPK model against literature reported DDIs for APAP mediated by other enzyme pathways besides SULTs. Nevertheless, the PBPK model analysis (Figure 4) illustrates that UGTS are responsible for only 20% of APAP metabolism in neonates. Although the contribution is much higher in infants and children, no single UGT pathway (1A1, 1A9, or 2B15) contributed to more than 19% metabolism, indicating that APAP systemic exposure changes would be minimal if one of these isozymes were modulated.

While these results are promising, certain limitations remain. First, the raw SULT enzyme abundance data used for ontogeny modeling included only postnatal (chronological) age, as post-menstrual age (PMA) was not available. Although PMA is a more developmentally appropriate measure in neonates and preterm infants, we were unable to reconstruct it for our analysis, which may have introduced some bias, particularly in the <2 year age group. Second, the ontogeny equations were derived using piecewise regression models based on the best statistical fit to individual data, resulting in phase boundaries that do not necessarily align with conventional pediatric age categories. This approach, however, was chosen to capture natural inflection points in enzyme development best, as supported by the raw data. Third, our modeling approach used mean enzyme abundance values and did not explicitly incorporate inter-individual variability, due to limitations in available sample size and data structure. As a result, the current model is best suited to evaluate age group–level trends in acetaminophen metabolism, rather than provide individualized predictions. Additionally, unlike some published studies including Ladumor et al., 2019 [7], our fitted ontogeny profiles derived from individual-level data do not display a pronounced decline in SULT abundance during late childhood (6–12 years). This difference may reflect the use of individual versus group mean data, age groupings, or the statistical modeling approach. Future research should prioritize the use of longitudinal data and virtual populations that include biological variability, genetic factors, and environmental influences to enable robust patient-level simulation and improve risk assessment. Additionally, integration of other metabolic pathways, such as glucuronidation and N-hydroxylation, could further enhance model robustness.

Despite these limitations, this study provides a physiologically grounded framework for evaluating SULT-mediated drug metabolism in pediatric populations. The incorporation of ontogeny-adjusted enzyme expression into PBPK models enhances our ability to simulate age-appropriate pharmacokinetics and optimize pediatric dosing. Future research should extend these efforts by validating models with in vivo data and integrating dynamic enzyme expression profiles to provide a comprehensive risk assessment. Our current focus is applying the PBPK model to characterize APAP disposition and guide dosing decisions in preterm babies undergoing treatment for patent ductus arteriosus.

Overall, this study underscores the crucial role of developmental physiology in pediatric pharmacokinetics and the value of PBPK modeling in informing safer and more effective pediatric drug therapy.

## 5. Conclusions

This study demonstrates the utility of integrating age-dependent SULT enzyme ontogeny into a PBPK model framework to predict APAP metabolism in pediatric populations. By accounting for developmental changes in enzyme expression, the models provide critical insights into drug metabolism in neonates and infants, where physiological immaturity necessitates individualized dosing strategies.

The PBPK model accurately predicted systemic exposure across pediatric age groups and aligned well with clinical data, reinforcing the pivotal role of SULT enzymes in early-life pharmacokinetics. Despite a moderate statistical fit due to biological variability, the ontogeny-informed framework reliably captured developmental trends and informed DDI risk in silico.

Notably, the simulations highlighted that neonates are more sensitive to enzyme inhibition or modulation than older children, emphasizing the need for age-specific consideration in DDI risk assessment.

In conclusion, this work advances the application of ontogeny-informed PBPK modeling in pediatrics, providing a framework for more accurate, safe, and effective dosing strategies in vulnerable populations. As pediatric pharmacology moves toward personalized medicine, the integration of developmental enzyme profiles will be crucial in optimizing drug therapy and minimizing adverse effects.

## Figures and Tables

**Table 1:** Input parameters to create acetaminophen compound profile in Simcyp^®^ [18].

Parameter ^a^	Value
**Physicochemical Properties**	
Molecular weight	151.2 g/mol
LogP	0.51
pKa	9.46
Fraction unbound in plasma	0.82 (Bound to albumin)
Blood/plasma ratio	1
**Absorption**	
Absorption Model	ADAM
P_eff_	12
ka	5.24
fa	0.99
**Dissolution**	
Solubility (mg/mL) at pH 8.94	13.65
**Distribution**	
Distribution Model	Minimal PBPK model
V_ss_ (L/kg) ^b^	0.8
Kp scalar ^b^	1.63
**Metabolism/Elimination**	
Clearance Type	Enzyme Kinetics
UGT 1A1	V_max_ = 6654; K_m_ = 5500
UGT1A9	V_max_ = 11,130; K_m_ = 9200
UGT2B15	V_max_ = 37,101; K_m_ = 23,000
SULT 1A1	V_max_ = 1549; K_m_ = 2400
SULT 1A3	V_max_ = 231.0; K_m_ = 1500
SULT1E1	V_max_ = 167.2; K_m_ = 1900
SULT2A1	V_max_ = 814.1; K_m_ = 3700
CYP1A2	V_max_ = 34.5; K_m_ = 220
CYP2C9	V_max_ = 9.86; K_m_ = 660
CYP2C19	V_max_ = 29.87 K_m_ = 2000
CYP2D6	V_max_ = 6.57; K_m_ = 440
CTP2E1	V_max_ = 90.06; K_m_ = 4020
CYP3A4	V_max_ = 62.13; K_m_ = 130
	
Cl_IV_ (L/h)	19.7
Cl_R_ (L/h)	1.12
Active hepatic scalar	1.5

^a^ Abbreviations: LogP: the logarithm of n-octanol, buffer partition coefficient, pKa; dissociation constant, ADAM: advanced dissolution; absorption and metabolism model, P_eff_: human jejunum effective permeability, fa: fraction of dose absorbed, ka: absorption rate constant, V_ss_: steady-state volume of distribution, Kp scalar: scalar applied to all predicted tissue partition values, CL_IV_: systemic clearance, Cl_R_: renal clearance, V_max_ (pmol/min/mg_protein_), K_m_ (uM). ^b^ published parameter estimate was modified in this research to optimize the model.

**Table 2:** Equations used to model SULT ontogeny.

Enzyme	Ontogeny Equation *	
**SULT1A1**	Abundance=107.8+88.6×Age	[age < 1.8 years]
	$Abundance=370.1+6.99\times \left(Age-1.83\right)$	[1.83 ≤ age ≤ 12.6 years]
	Abundance=445.2	[age > 12.6 years]
**SULT1A3**	Abundance=117.3−28.7×Age	[age < 2 years]
	$Abundance=60+5.66\times \left(Age-2\right)$	[2 ≤ age ≤ 8.6 years]
	Abundance=97.2	[age > 8.6 years]
**SULT2A1**	Abundance=705.3+975.2×Age	[age < 1.2 years]
	$Abundance=1893-65.5\times \left(Age-1.2\right)$	[1.2 ≤ age ≤ 13.2 years]
	Abundance=1095	[age > 13.2 years]
**SULT1B1**	Abundance=45.5+37.3×Age	[age < 2.6 years]
	$Abundance=140.6-4.69\times \left(Age-2.6\right)$	[2.6 ≤ age ≤ 8.1 years]
	Abundance=115	[age > 8.1 years]

* Best fit equation to predict enzyme abundance (pmol/mg_cytosolic protein_) as a function of age (years).

**Table 3:** Acetaminophen PBPK model verification in adult Healthy Volunteers.

Dose	Parameter	PBPK Model-Predicted	Observed *	Fold Error
5 mg/kg Infusion	C_max_ (μg/mL)	4.95 ± 0.57	4.74	1.04
	AUC_0-∞_ (µg-hr/mL)	22.9 ± 6.15	18.4 ± 1.65	1.24
20 mg/kg Infusion	C_max_ (μg/mL)	20.0 ± 2.27	17.8	1.12
	AUC_0-∞_ (µg-hr/mL)	93.5 ± 25.3	82.5 ± 10.0	1.13

* Presented as mean ± standard deviation. Standard deviation was not reported for observed Cmax values. Only the mean is presented. Reference: Clements 1984 [19].

**Table 4:** Acetaminophen PBPK model verification in neonates, infants, and children.

Population (Dosing Regimen)	Parameter *	PBPK Model Predicted	Observed	Fold Error
Children (12.5 mg/kg Q 6h)	C_max_ (μg/mL)	28.6 (23.9–40.2)	24.3 (3.8–35.1)	1.18
	AUC_0-τ_ (µg-hr/mL)	35.7 (20.7–76.4)	37.8 (11.3–52.3)	0.94
Infants (12.5 mg/kg Q 4h)	C_max_ (μg/mL)	22.8 (18.4–30.2)	21.9 (4.2–25.3)	1.04
	AUC_0-τ_ (µg-hr/mL)	39.1 (22.7–69.3)	43.3 (9.2–79.2)	0.90
Neonates (12.5 mg/kg Q 4h)	C_max_ (μg/mL)	21.8 (17.7–28.5)	19.9 (19.3–20.5)	1.10
	AUC_0-τ_ (µg-hr/mL)	53.6 (26.7–92.1)	65.6 (55.8–75.4)	0.82

* Presented as median (range). Reference: Zuppa 2011 [21]

## Data Availability

The data presented in this study are available on request from the corresponding author.

## Supplementary Materials

The following supporting information can be downloaded at: https://www.mdpi.com/article/10.3390/life15071099/s1, Figure S1. Sensitivity analysis illustrating the impact of changes in Michaelis-Menten parameters (Km, Vmax) on acetaminophen Cmax Ratio in neonates, infants, and children.
