# Supplementary material for: PBPK Modeling of Acetaminophen in Pediatric Populations: Incorporation of SULT Enzyme Ontogeny to Predict Age-Dependent Metabolism and Systemic Exposure
Source: Life (Basel). 2025 Jul 13;15(7):1099. doi: 10.3390/life15071099 (PMC12300260; doi:10.3390/life15071099)
Supplement: Supplementary file 1 [file life-15-01099-s001.zip › life-3707947-supplementary.pdf]

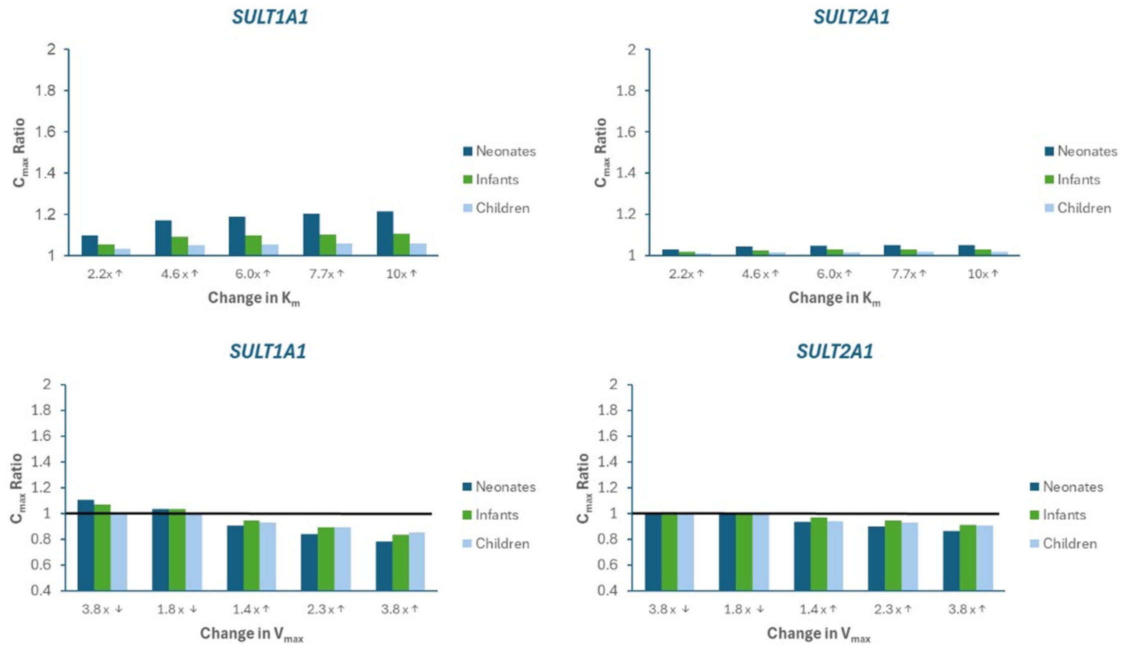

Figure S1. Sensitivity analysis illustrating the impact of changes in Michaelis-Menten parameters (Km, Vmax) on acetaminophen C_{max} Ratio in neonates, infants, and children. C_{max} Ratio is calculated as the predicted AUC relative to baseline model values.
